# Health-Related Quality of Life and Costs of Posttraumatic Stress Disorder in Adolescents and Young Adults in Germany

**DOI:** 10.3389/fpsyt.2020.00697

**Published:** 2020-07-15

**Authors:** Judith Dams, Eline Rimane, Regina Steil, Babette Renneberg, Rita Rosner, Hans-Helmut König

**Affiliations:** ^1^Hamburg Center for Health Economics (HCHE), Department of Health Economics and Health Services Research, University Medical Center Hamburg-Eppendorf, Hamburg, Germany; ^2^Department of Psychology, Catholic University Eichstätt-Ingolstadt, Eichstätt, Germany; ^3^Department of Clinical Psychology and Psychotherapy, Institute of Psychology, Goethe University Frankfurt, Frankfurt, Germany; ^4^Department of Clinical Psychology and Psychotherapy, Freie Universitaet Berlin, Berlin, Germany

**Keywords:** cost-of-illness, posttraumatic stress disorder, quality of life, economic burden, adolescents

## Abstract

**Background:**

Posttraumatic stress disorder (PTSD) is one of the psychopathological consequences of sexual and/or physical abuse. The economic burden is assumed to be high, whereas health-related quality of life and education is negatively affected. This study aims to determine health care costs, health-related quality of life, and educational interruption in adolescents and young adults with PTSD after sexual and/or physical abuse in Germany.

**Methods:**

This analysis used data of 87 participants aged 14–21 years of a randomized controlled trial. Health care utilization, health-related quality of life (EQ-5D-5L), sick leave days, productivity, and delay or failure to achieve educational aims were assessed. Health care costs from a payer perspective were calculated using unit costs for the year 2014.

**Results:**

Mean health care costs for a six-month period were 5,243€ (SE 868€). In particular, costs of inpatient stays in psychiatric hospitals, general hospitals and rehabilitation as well as child welfare institutions were high. In addition, health-related quality of life was lower due to anxiety/depression, resulting in a mean EQ-5D index and EQ-VAS score of 0.70 and 61.0, respectively. Furthermore, participants reported on average 27 sick leave days, a productivity loss of 61%, and a delay in education attainment as well as having been unable to achieve educational aims.

**Conclusion:**

PTSD in adolescents and young adults is associated with a high economic burden. Health-related quality of life was substantially reduced. Furthermore, delay and productivity losses in education were observed.

**Clinical Trial Registration:**

German Clinical Trials Register identifier: DRKS00004787; date of registration: 18^th^ March 2013; https://www.drks.de.

## Introduction

Posttraumatic stress disorder (PTSD) is characterised by distress symptoms lasting more than one month following a traumatic event, such as sexual and/or physical abuse, accidents or war experiences. According to DSM-IV, PTSD is characterised by intrusive reliving of the traumatic events, avoidance of reminders of the trauma, and hyperarousal symptoms. Furthermore, patients have to suffer from clinical distress, or experience difficulties in social or occupational functioning to be diagnosed with PTSD (1).

The high lifetime prevalence of PTSD has been estimated by numerous studies (2, 3). As it has been estimated that 30% to 40% of children after sexual or physical abuse develop PTSD (4), PTSD in adolescents and young adults is rather frequent.

Patients often suffer from long-term consequences such as mental and somatic comorbidities, unemployment and reduced productivity as well as reduced quality of life (5–8). Moreover, the consequence of PTSD, with respect to health care costs (direct costs), impairments in working life (indirect costs) and constraints on quality of life, can be significant (9).

International treatment guidelines as the one by the National Institute for Health and Care Excellence (NICE) recommend trauma-focused cognitive behavioural therapy or eye movement desensitization and reprocessing therapy (10). Based on the results in adults, Matulis et al. adapted Cognitive Processing Therapy (CPT) (11) to the needs of adolescents and young adults following abuse. Developmentally Adapted Cognitive Processing (D-CPT) was introduced, which integrated four major adjustments of CPT tailored specifically to adolescent survivors of childhood abuse suffering from PTSD (11). To facilitate the motivation of adolescents to participate in treatment, a commitment phase was integrated and the frequency of treatment sessions was increased. Furthermore, behaviour and emotion management techniques suitable for adolescents were included. Specifically, the therapy focuses on difficulties in emotion regulation, which often occur after sexual or physical childhood abuse. Finally, developmental tasks tailored to the situation of each patient were established. For example, a goal may seek to minimise the risk of patients of future revictimization and/or the risk of patients selecting an abusive partner.

Even though the consequences of PTSD are significant, only a limited number of health economic studies addressing PTSD after sexual abuse exist (7, 12–16). Most of these studies analyzed data of adult patients with PTSD (12–14). The economic burden was reported to be high. Annual costs ranged between 3,060 US$ and 18,100€ per adult patient. Costs due to psychiatric treatment were reported to be particularly high in a German study of adults with PTSD (12). However, in younger age groups, PTSD may have a specific impact on the utilisation of health care and health-related quality of life, as well as on the educational development of patients.

To our knowledge, only one empirical health economics study that focusses on cost of illness of adolescents and young adults with PTSD after sexual and/or physical childhood abuse in the UK has been published (16). This study was an 11-week randomised controlled trial, thus long-term effects were estimated using a Markov model. Even though indirect costs might not be relevant in patients at a young age (as they do not participate in the labour market yet), educational interruption may have an influence on future indirect costs. Several studies evaluated by a review (17) have addressed the effects of traumatic experience on school performance. However, this review focused on adolescents or young adults with maltreatment, rather than on adolescents after sexual and/or physical childhood abuse. Thus, these effects on school performance are still unknown. Beyond costs, the burden of PTSD may be described by the impact of PTSD on patients’ health-related quality of life. In health economic research, the EQ-5D is the most frequently used generic quality of life questionnaire. To our knowledge, health-related quality of life of adolescents and young adults with PTSD measured by the EQ-5D has not yet been published in the literature.

Therefore, this study estimated the economic consequences of PTSD with respect to health care utilisation, costs and health-related quality of life for adolescents and young adults with PTSD in Germany. In addition, it determined the impact of PTSD on educational interruption, which could be considered as an indicator of future indirect costs.

## Methods

### Study Design and Participants

This study used baseline data on health care utilisation and health-related quality of life of participants enrolled in a multicentre randomized controlled trial (RCT) for adolescents and young adults with PTSD (18, 19). Study participants were recruited through therapists, psychiatric clinics and youth welfare institutions as well as flyers and press releases. Screening, assessment and therapy took place at German outpatient university clinics in Berlin, Frankfurt/Main, and Eichstätt/Ingolstadt. Participants were enrolled between July 23, 2013 and June 17, 2015. Informed consent was obtained from the participants and if participants were 18 years old or less, informed consent was obtained from their parents or legal guardians.

Participants with a primary diagnosis of PTSD related to childhood sexual and/or physical abuse were eligible to participate, provided they were also between the ages of 14 and 21, had no or a stable psychopharmacological medication (for ≥ 3 weeks) and lived in stable conditions. Furthermore, sufficient knowledge of German language was required. Participants with current severe or life-threatening suicidality or self-harming behaviour within the last 6 month, IQ ≤ 75, current substance dependence (abstinence < 6 months), or a substance-induced disorder, any documented pervasive developmental disorder, lifetime psychotic or bipolar disorder (unclear cases were included) according to the DSM-IV-TR (1), as well as simultaneous psychotherapeutic treatment, were excluded. Participants were randomized to treatment with D-CPT or wait-list condition with treatment advice (WL/TA)). Participants with at least two avoidance symptoms in the clinical interview defined in the DSM-IV-TR (1) were diagnosed with PTSD. Analyses were based on data from the included participants with information on health care utilisation at baseline (n=87). A detailed description of study design and treatment options as well as results in clinical outcomes can be found elsewhere (18, 19).

### Socio-Demographics and Clinical Parameters

Socio-demographics included individual characteristics (e.g., age, gender, migration status) as well as family background (number of siblings, parental marital status), living situation, education and educational level of the parents. Clinical parameters included information on previous treatment and the severity of PTSD as measured by the Clinician Administered PTSD Scale for Children and Adolescents (CAPS-CA) (20, 21). The CAPS-CA rates frequency and intensity of PTSD symptoms from 0 (never/no problem) to 4 (most of the time/extreme problem), with a total score ranging from 0 (best) to 136 (worst). In addition, participants were asked to complete a wide range of questionnaires to determine the presence and severity of specific psychiatric comorbidities (e.g., Structured Clinical Interview for DSM Disorders (SCID) (22–24), the Diagnostic Interview of Mental Disorders in Childhood and Adolescence (Kinder-DIPS) (25), the nicotine section of the Expert System for Diagnosing Mental Disorders (DIA-X) (26).

### Health Care Costs

Health care utilisation during the six months prior to baseline was assessed using an adapted version of the Client Socio-demographic and Service Receipt Inventory (CSSRI) questionnaire (27). Utilisation of health care was monetarily valued using German unit costs for each health care category for the year 2014 (**Supplementary Material, Table S1**) (28). We calculated health care costs from a payer perspective. Costs included stays in general hospitals, psychiatric hospitals, or rehabilitation clinics, outpatient physician and non-physician (e.g., occupational therapy, physiotherapy) services as well as medication. Medication costs were derived from the German official pharmaceutical index (Rote Liste) (29). Furthermore, the services of youth welfare, social counselling, and child welfare institutions (living in a foster family or in an apartment with other adolescents, assisted by social workers) were included.

### Health-Related Quality of Life

Health-related quality of life was assessed by the EQ-5D-5L (30, 31) which records problems across the dimensions of “mobility”, “self-care”, “usual activities”, “pain/discomfort”, and “anxiety/depression”. Participants rate their experience on an ordinal scale with “no problems”, “slight problems”, “moderate problems”, “severe problems” to “extreme problems”. As persons filling in the EQ-5D-5L are asked to choose one out of five levels for each of the five dimensions, the EQ-5D-5L descriptive system describes 3125 (5^5^) different health states, which could be transferred to the EQ-5D index. The EQ-5D index is a score between -0.661 (extreme problems) and 1 (best possible health) (32). In addition, quality of life was measured by the visual analogue scale of the EQ-5D (EQ-VAS) on a scale between 0 (worst) and 100 (best) (30).

### Education

Educational interruptions were assessed by questions concerning school education and professional training, including the number of days absent from school/professional training due to PTSD over the previous six months. Even if patients had participated at school/professional training, productivity loss might have occurred. Therefore, productivity loss at school/professional training due to PTSD was considered using a visual analogue scale ranging from 0 (“no productivity loss”) to 10 (“complete productivity loss”). In addition, educational delays due to PTSD were assessed by asking for repeated years at school. Furthermore, participants reported failed school graduation or professional training graduation due to PTSD. Finally, problems in finding a professional training position or job were evaluated by a visual analogue scale between 0 (“no problems”) and 10 (“severe problems”). As most participants did not complete school yet, educational interruptions and productivity loss at school were not monetarily valued.

### Statistical Analysis

The maximum missing rate per variable in the evaluated data set was 23% for sick leave days at school. Of all participants, 10% did not report their achieved educational level or their health care utilisation for inpatient stays in general hospitals and rehabilitation. In 5% to 10% of all cases information on child welfare institutions, outpatient physician visits, counselling, and inpatient stays in psychiatric hospitals was missing. The proportions of missing values for all other variables were 5% or less. Even though no common threshold for an inconsequential proportion of missing data is reported in the literature, the use of imputation methods for missing rates above 5% is recommended (33). As the missing rate for some essential variables (sick leave days, inpatient stays in general hospitals and rehabilitation) in the current data were above 5%, missing values were imputed to avoid a loss in statistical power. We used multiple imputation by chained equations (MICE) with predictive mean matching to create 50 imputations (33, 34).

We analyzed costs, health-related quality of life and variables on education/professional training using descriptive statistics, and determined the influence of age, gender, CAPS-CA total score, and psychiatric comorbidities on costs and health-related quality of life to identify subgroups of patients with increased and decreased costs or health-related quality of life. For total cost data generalized linear models (GLMs) with a Gamma distribution and a log-link function were used, whereas health-related quality of life was analyzed using linear regression. For all analyses, a significance level of 5% was applied.

Subgroup analyses determined costs by study centres and treatment options. Differences between subgroups were tested using regression analyses adjusted for age, gender, CAPS-CA total score, and psychiatric comorbidities.

All computations were conducted with R (3.5.1). In particular, the R-package “mice” was used for multiple imputation (35).

## Results

### Sample Characteristics

Participants had a mean age of 18.1 (SD 2.3) years, and 15% were male (**Table 1**). The mean CAPS-CA total score was 64.7 (SD 21.7). On average participants had 1.9 (SD 1.5) psychiatric comorbidities. Prior to inclusion, 61% of the participants had undergone outpatient psychotherapy and 44% had at least one psychiatric hospital stay.

**Table 1:** Sample characteristics (n=87).

Demographics			
*age*	mean | SD	18.1	2.3
*male gender*	n | %	13	14.9
secondary school degree or higher	n | %	11	12.6
number of siblings	mean | SD	2.3	2.0
*Severity of disease*			
CAPS-CA total score	mean | SD	64.7	21.7
*Comorbidities*			
number of psychiatric comorbidities	mean | SD	1.9	1.5
*Previous therapy*			
previous outpatient psychotherapies/psychiatric therapies >=1	n | %	53	60.9
previous inpatient psychotherapies/psychiatric therapies >=1	n | %	38	43.7

CAPS-CA, Clinician Administered PTSD Scale for Children and Adolescents.

### Costs

Mean total costs were 5,243€ (SE 868) for the 6-month prior to baseline (**Table 2**). Of these costs, 50% were due to inpatient psychiatric hospitals (1,198€, SE 510), general hospitals (936€, SE 217), and rehabilitation hospitals (450€, SE 204). Half of the costs were due to outpatient services, in particular child welfare institutions (1,638€, SE 483), and outpatient physician treatment (696€, SE 80). Neither gender, the CAPS-CA total score nor the number of psychiatric comorbidities were related significantly to costs (**Table 3**). Unadjusted subgroup analyses revealed differences in costs due to study centre and treatment options (**Supplementary Material, Tables S2** and **S3**). However, after adjusting for sociodemographic and clinical characteristics, no statistically significant group differences were observed.

**Table 2:** Six-month costs in 2014 Euros (n=87).

	mean	median	SE	range	% of total costs	% of the sample with utilization
general hospital	936	0	217	0-8,220	18	18
psychiatric hospital	1,198	0	510	0-29,095	23	8
rehabilitation	450	0	204	0-12,175	9	6
outpatient physician treatment	696	483	80	0-3,824	13	97
psychotherapy/psychiatric/neurological	401	159	66	0-3,582	8	53
outpatient non-physician treatment	20	0	11	0-765	0	7
medication	58	0	13	0-727	1	46
child welfare institutions	1,638	0	483	0-14,396	31	16
counselling	247	0	70	0-4,529	5	38
**total costs**	**5,243**	**949**	**868**	**20-21,243**	**100**	**100**

**Table 3:** Determinants of costs (n=87).

Parameters	coefficient	SE	*p*-value	95% CI	
age	0.94	0.08	0.39	0.80	1.09
gender (female=0, male=1)	1.46	0.48	0.43	0.56	3.78
CAPS-CA total score	1.00	0.01	0.79	0.98	1.02
number of psychiatric comorbidities	1.06	0.13	0.63	0.82	1.37
intercept	16,240	1.49	0.00	842	313,180

CAPS-CA, Clinician Administered PTSD Scale for Children and Adolescents.

### Health-Related Quality of Life

Health-related quality of life as measured by the EQ-5D-5L was most strongly affected by the dimension of anxiety/depression with 13% of the participants reporting extreme, 22% severe, and 35% moderate problems (**Table 4**). Furthermore, participants frequently reported problems in the dimension of pain/discomfort, with 13% reporting severe or extreme problems and 32% moderate problems. Less problems were reported in the dimensions of mobility and usual activities. Furthermore, participants were rarely impaired with respect to the dimension of self-care. The mean EQ-5D index and EQ-VAS score were 0.70 (SE 0.03) and 61.0 (SE 2.4), respectively.

**Table 4:** EQ-5D-5L (n=87).

	n (%) of participants with problems				
EQ-5D-5L dimensions	“no”	“slight”	“moderate”	“severe”	“extreme”
mobility	62 (71)	17 (20)	7 (8)	1 (1)	0 (0)
self-care	82 (94)	3 (4)	0 (0)	2 (2)	0 (0)
usual activities	37 (43)	23 (26)	16 (18)	6 (7)	5 (6)
pain/discomfort	23 (26)	25 (29)	28 (32)	8 (9)	3 (4)
anxiety/depression	9 (10)	17 (20)	31 (35)	19 (22)	11 (13)

EQ-5D-5L, Euro-QoL- 5 dimensions – 5 levels.

Analysis on determinants of health-related quality of life revealed that the EQ-5D index was not statistically significantly different between males and females. Furthermore with increasing CAPS-CA total score the EQ-5D index was significantly reduced (**Table 5**). Neither gender, the CAPS-CA total score nor the number of psychiatric comorbidities showed any significant relation to the EQ-VAS.

**Table 5:** Determinants of health-related quality of life measured by the EQ-VAS and EQ-5D index (n=87).

parameters	EQ-VAS					EQ-5D index				
	coefficient	SE	*p*-value	95% CI		coefficient	SE	p-value	95%CI	
age	-0.52	1.02	0.61	-2.54	1.50	0.00	0.01	1.00	-0.02	0.02
gender (female=0, male=1)	3.03	6.34	0.63	-9.58	15.64	-0.10	0.07	0.14	-0.23	0.04
CAPS-CA total score	-0.23	0.12	0.05	-0.46	0.00	-0.01	0.00	0.00	-0.01	0.00
number of psychiatric comorbidities	-3.00	1.64	0.07	-6.27	0.27	-0.00	0.07	0.99	-0.04	0.03
intercept	91	20	0.00	52	130	1.12	0.21	0.00	0.71	1.53

SE, standard error; CI, confidence intervals; EQ-VAS, visual analogue scale of the EQ-5D; CAPS-CA, Clinician Administered PTSD Scale for Children and Adolescents.

### Education

Participants reported on average 27 sick-leave days in school/professional training in the prior six months. Furthermore, a productivity loss of 61% was measured. Long-term effects on education were appraised according to delays in achieving educational aims. Overall, 24 participants had repeated a year in school. Of those, 30% repeated the year at the end of secondary school. Furthermore, 25 participants reported to have failed school graduation or professional training graduation due to PTSD. Even though only 42 adolescent participants had completed their school/professional training, 20 reported being unable to pursue their preferred job. Furthermore, these participants rated problems in finding a professional training or job to be 1.5 on scale between 0 (“no problems”) and 10 (“severe problems”).

## Discussion

Our analyses revealed a high burden of direct costs, reduced health-related quality of life and educational interruption among adolescents and young adults with PTSD.

### Costs

Costs were mainly due to psychiatric hospitals, general hospitals and rehabilitation. As most of the participants had at least one psychiatric comorbidity, inpatient costs may be due to these. In addition, high costs were due to the utilisation of child welfare institutions. Of all participants, 15% were living neither at home nor with relatives/friends. The trigger of the disease may be the reason, as it is often located in the family environment. Almost every participant used outpatient physician treatment. Yet, compared to inpatient treatment costs, only moderate outpatient treatment costs were observed. More than half of the outpatient treatment costs were due to psychological treatment.

To our knowledge, one study has estimated cost of PTSD in children and adolescents (16) and only two studies have estimated costs of PTSD in children (7, 15). The first cost-utility analysis was based on an 11-week trial, which revealed health care costs of 557 £ in the UK (16). In accordance with our results inpatient costs and community services were high. Another study estimated intervention costs for three treatment strategies for Australian children with PTSD after sexual abuse (7). In this study, costs were not measured empirically but were estimated based on expert opinion. Intervention costs of cognitive behaviour therapy (CBT), CBT combined with selective serotonin reuptake inhibitor, and non-directive counselling were compared. Over a 12-month period intervention costs of 2,074 AUD for non-directive counselling, 2,051 AUD for CBT only, and 2,226 AUD for CBT combined with selective serotonin reuptake inhibitor were calculated. Yet, only intervention costs were included. The last trial considered children with PTSD in the USA, however the study did not specifically pertain to participants who had experiences of sexual abuse (15). Mental health care costs including costs for crisis centres, counselling and community support, and medical supervision for participants treated with CBT were compared with services-as-usual. Total annual costs were 3,055 USD for participants treated with CBT and 4,208 USD for participants receiving services-as-usual. In particular, outpatient costs were high compared with our results, respectively. Higher costs compared with our results were expected due to the treatment with CBT or services-as-usual, whereas our study included participants pre-treatment. Yet, data did not include all relevant costs categories (15). Costs of medication, counselling or child welfare institutions were not considered.

Unfortunately, no cost data for young patients with PTSD or a representative sample of adolescents and young adults of the general population in Germany have been published. One study reported costs for the German health care system for adult with PTSD (12). In line with our results, high 6-month costs for psychiatric inpatient (7,596€) and outpatient (825€) treatment were observed. Yet, our results for psychiatric inpatient costs in adolescents and young adults were lower (1,198€ for 6 months). Specifically, adolescents spent fewer days in psychiatric hospitals compared with adults (3 vs. 45 days), which was expected as adolescents were offered outpatient treatment, whereas adults were treated in inpatient settings. In addition, costs for outpatient psychotherapy were more than twice as high in adults compared with our results (825€ vs. 401€) which in addition to the treatment location may be explained by fewer comorbidities due to the younger age.

### Health-Related Quality of Life

Health-related quality of life in adolescents and young adults with PTSD was markedly reduced. Participants indicated a mean EQ-5D Index and EQ-VAS score of 0.70 and 61.0, respectively. Our results showed considerable impairments for participants with PTSD, when compared with a representative German population sample aged between 8 and 18 years (35). Persons of the general population reported an average EQ-VAS score of 83.5.

On the EQ-5D-5L dimension anxiety/depression, 13% and 22% of the participants reported extreme and severe problems, whereas only 2% in the general populations were impaired extremely or severely. Furthermore, 90% of the participants with PTSD reported to have any problems due to anxiety/depression, which was much more frequent compared with only 22% of the general population. In addition to problems due to anxiety/depression, our results indicated problems of adolescents and young adults with PTSD in the EQ-5D-5L dimension of pain/discomfort. Whilst only 2% and 26% of the general population reported to have extreme/severe and slight/moderate problems respectively, 13% and 61% of the adolescents and young adults with PTSD reported such problems. Furthermore, participants with PTSD reported more problems in the dimensions of mobility and usual activities, although less frequently than in the dimensions of anxiety/depression and pain/discomfort. Problems with usual activities were rated to be extreme/severe or slight/moderate by 13% and 44% of the participants, whereas only 1% and 12% of the persons of the general population reported such problems. Furthermore, 28% of the participants with PTSD experienced slight/moderate problems in the dimension of mobility compared to 4% in the general population. Both problems due to mobility, as well as usual activities may be due to the desire among those diagnosed with PTSD to avoid the re-experience of the traumatic event and/or the hyperarousal caused by the traumatic event. For both, persons with PTSD and the general population, only few problems were reported on the dimensions self-care, where more than 94% experienced no problems. In conclusion, health-related quality of life was decreased among adolescents and young adults with PTSD. In particular, anxiety/depression seems to have a substantial influence.

To our knowledge only one other studies reported health-related quality of life for children with PTSD aged between 9 and 11 years (17). Participants reported mild to moderate impairments on the Life Satisfaction Scale, thus our findings confirm previous results.

### Education

During a six-month period, adolescents and young adults with PTSD had on average 27 sick leave days in school/professional training. Productivity was reduced by 61%.

In line with our results, a recently published systematic review considering the impact of traumatic experiences on school performances revealed a reduced school performance of participants with PTSD (36). Furthermore, studies reported a delay in educational attainment by repeating a year at school (37–40). To compare our results with data of the German population we used data from the federal statistical office (41). The mean probability of repeating one specific year at school was 1.33%. Thus, for n=87 participants and an assumed 11-year school education at the age of 18 years, we would expected 15 participants to have repeated one year. Indeed, 24 adolescents and young adults with PTSD repeated one year.

Furthermore, adolescents and young adults with PTSD had achieved a lower educational level compared with the German population (41). Specifically, participants with PTSD less frequently completed a technical college or an A-level exam. As most participants had not completed school, our results may not be directly comparable with data from the federal statistical office. However, 42 participants had completed their school/professional training, and half of them were unable to start or complete their preferred job.

### Strength and Limitations

Even though PTSD is highly prevalent and has significant consequences, only a small number of studies have examined its effects on costs, health-related quality of life and education in adolescents and young adults. Although the estimated number of unreported cases of abuse-related PTSD is high, we were able to provide empirical data of 87 adolescents and young adults with PTSD to close this gap in the literature. The evaluation of resource utilisation was adapted to the study population, and the costs associated with the utilisation of the established CSSRI youth welfare services, social counselling and child welfare institutions were also assessed. Costs due to transport and long-term nursing care were not assessed. Instead of indirect costs due to absenteeism from work and productivity loss, performance of adolescents and young adults with PTSD at school/professional training were evaluated. Finally, we used advanced statistical methods. Missing values were handled by multiple imputation using MICE. MICE is widely used to impute values missing (completely) at random, thus a loss of statistical power was avoided. In addition, the skewness in cost data was taken into account using GLMs assuming a Gamma distribution and log-link function.

Even though the study was carried out carefully, it does include some limitations. First, the sample size of the study (n=87) was rather small. As the distribution of cost data was skewed, the small sample size may have led to a bias in cost calculations. Nevertheless, results showed a reduced quality of life and high costs for adolescents and young adults with PTSD. Second, multiple imputation using MICE requires missing at random (MAR). Unfortunately, no statistical test exists to distinguish between MAR and missing not at random (MNAR), thus MNAR could not be ruled out. However, sociodemographic statistics for persons, who did not report health care utilization in major cost drivers (psychiatric hospital, general hospital, outpatient non-physician treatment, child welfare institutions), did not differ compared with those of the complete sample. Finally, almost half of the participants had not completed school yet. Therefore, results on educational interruption cannot be interpreted as long-term consequences of PTSD. In addition, further problems may arise in later working life (e.g., when trying to find a job). Therefore, future research should consider long-term economic consequences of PTSD in adults after sexual and/or physical abuse.

Finally, costs were collected independent of seasons for 6 months prior to baseline, so that the influence of seasons on health care utilization could not be determined. However, a seasonal influence on the results was not expected, because no seasonal clinical events are known for PTSD.

## Conclusion

PTSD in adolescent and young adults has a high economic burden. High costs due to inpatient stays in psychiatric hospitals, general hospitals and rehabilitation as well as child welfare institutions occurred. Health-related quality of life was substantially reduced. Furthermore, considerable delays and productivity losses in education were observed.

## Data Availability Statement

The datasets presented in this article are not readily available because of ethical and confidentiality concerns.

## Ethics Statement

The studies involving human participants were reviewed and approved by Ethics Committee of Catholic University Eichstätt-Ingolstadt, Eichstätt, Germany; the Freie Universitaet of Berlin,Berlin, Germany; and the Goethe University Frankfurt, Frankfurt am Main, Germany. Written informed consent was obtained from all participants and in the case of minors from the participants' legal guardian.

## Author Contributions

ER, RS, BR, RR, and H-HK conceived the study and developed the design. JD was responsible for the analysis and wrote the first draft. All authors contributed to the article and approved the submitted version.

## Funding

This study was supported by grants from the German Ministry of Education and Research (grant numbers 01KR1204D, 01KR1204A, and 01KR1204C). The sponsor had no role in the design and conduct of the study; collection, management, analysis, and interpretation of the data; preparation, review, or approval of the manuscript; and decision to submit the manuscript for publication.

## Conflict of Interest

RR reported being paid fees for workshops and presentations on posttraumatic stress disorder (PTSD) treatment and coauthoring a book on cognitive processing therapy. RS reported being paid fees for workshops and presentations on PTSD treatment.

The remaining authors declare that the research was conducted in the absence of any commercial or financial relationships that could be construed as a potential conflict of interest.

## References

[1] APA (American Psychiatric Assosication) Diagnostisches und Statistisches Manual Psychischer Störungen - Textversion - DSM-IV-TR. Sass H Wittchen HU Zaudig M Houben I, editors. Göttingen: Hogrefe (2003).

[2] Gilbert R Widom CS Browne K Fergusson D Webb E Janson S Burden and consequences of child maltreatment in high-income countries. Lancet (London England) (2009) 373(9657):68–81. 10.1016/s0140-6736(08)61706-7 19056114

[3] Sethi D Bellis M Hughes K Gilbert R Mitis F Galea G European report on preventing child maltreatment. Copenhagen, Denmark: World Health Organization, Regional Office for Europe (2013).

[4] Cutajar MC Mullen PE Ogloff JR Thomas SD Wells DL Spataro J Psychopathology in a large cohort of sexually abused children followed up to 43 years. Child Abuse Negl (2010) 34(11):813–22. 10.1016/j.chiabu.2010.04.004 20888636

[5] Ferry FR Bolton D Bunting BP O´Neill SM Murphy S Devine B The Economic Impact of Post Traumatic Stress Disorder in Northern Ireland. A report on the direct and indirect health economic costs of post traumatic stress disorder and the specific impact of conflict in Northern Ireland. Northern Ireland: Bamford Centre for Mental health and Wellbeing and the Northern Ireland Centre for Trauma and Transformation Trust (2012).

[6] Ferry FR Brady SE Bunting BP Murphy SD Bolton D O´Neill sM. The Economic Burden of PTSD in Northern Ireland. J Traumat Stress (2015) 28:191–7. 10.1002/jts.22008 25990825

[7] Gospodarevskaya E Segal L Cost-utility analysis of different treatments for post-traumatic stress disorder in sexually abused children. Child Adolesc Psychiatry Ment Health (2012) 6:15. 10.1186/1753-2000-6-15 22490433 PMC3383488

[8] Mihalopoulos C Magnus A Lal A Dell L Forbes D Phelps A Is implementation of the 2013 Australian treatment guidelines for posttraumatic stress disorder costeffective compared to current practice? A cost-utility analysis using QALYs and DALYs. Aust New Z J Psychiatry (2014) 49(4):360–76. 10.1177/0004867414553948 25348698

[9] Seedat S Lochner C Vythilingum B Stein DJ Disability and quality of life in post-traumatic stress disorder: impact of drug treatment. PharmacoEconomics (2006) 24(10):989–98. 10.2165/00019053-200624100-00006 17002481

[10] National Institute for Health and Care Excellence Post-traumatic stress disorder. NICE guideline. London: National Institute for Health and Care Excellence (2018).

[11] Matulis S Resick PA Rosner R Steil R Developmentally adapted cognitive processing therapy for adolescents suffering from posttraumatic stress disorder after childhood sexual or physical abuse: a pilot study. Clin Child Family Psychol Rev (2014) 17(2):173–90. 10.1007/s10567-013-0156-9 24101403

[12] Priebe K Roth M Krüger A Glöckner-Fink K Dyer A Steil R Psychiatrische Behandlungskosten von Patientinnen mit Posttraumatischer Belastungsstörung nach sexuellem Missbrauch vor und nach stationärer DBT-PTSD. Psychiatr Praxis (2016) 44(2):75–84. 10.1055/s-0042-106068 27399595

[13] Surís A Lind L Kashner M Borman PD Petty F Sexual Assault in Women Veterans: An Examination of PTSD Risk, Health Care Utilization, and Cost of Care. Psychosomatic Med (2004) 66:749–56. 10.1097/01.psy.0000138117.58559.7b 15385701

[14] Walker EA Katon W Russo J Ctechanowski P Newman E Wagner AW Sexual Assault in Women Veterans: An Examination of PTSD Risk, Health Care Utilization, and Cost of Care. Arch Gen Psychiatry (2003) 60:369–74. 10.1001/archpsyc.60.4.369 12695314

[15] Greer D Grasso DJ Cohen A Webb C Trauma-focused treatment in a state system of care: is it worth the cost? Adm Policy Ment Health (2014) 41(3):317–23. 10.1007/s10488-013-0468-6 23334468

[16] Shearer J Papanikolaou N Meiser-Stedman R McKinnon A Dalgleish T Smith P Cost-effectiveness of cognitive therapy as an early intervention for post-traumatic stress disorder in children and adolescents: a trial based evaluation and model. J Child Psychol Psychiatry Allied Discipl (2018) 59(7):773–80. 10.1111/jcpp.12851 PMC6597355 29197091

[17] Gillies D Taylor F Gray C O’Brien L D’Abrew N Psychological therapies for the treatment of post-traumatic stress disorder in children and adolescents. Cochrane Database Syst Rev (2012) 12:Cd006726. 10.1002/14651858.CD006726.pub2 23235632 PMC12206555

[18] Rosner R Konig HH Neuner F Schmidt U Steil R Developmentally adapted cognitive processing therapy for adolescents and young adults with PTSD symptoms after physical and sexual abuse: study protocol for a randomized controlled trial. Trials (2014) 15:195. 10.1186/1745-6215-15-195 24886027 PMC4055428

[19] Rosner R Rimane E Frick U Gutermann J Hagl M Renneberg B Effect of Developmentally Adapted Cognitive Processing Therapy for Youth With Symptoms of Posttraumatic Stress Disorder After Childhood Sexual and Physical Abuse: A Randomized Clinical Trial. JAMA Psychiatry (2019) 76(5):484–91. 10.1001/jamapsychiatry.2018.4349 PMC6495346 30969342

[20] Nader K Kriegler J Blake D Pynoos R Clinical Administered PTSD Scale, Child and Adoloescent Version (CAPS-C). White River Junction, VT: National Center for PTSD (1994).

[21] Steil R Füchsel G IBS-KJ. Interviews zu Belastungsstörungen bei Kindern und Jugendlichen. Diagnostik der aktuen und der posttraumatischen Belastungsstörung. Göttingen: Hogrefe (2006).

[22] First M Spitzer R Gibbon M Williams J Benjamin L Structured Clinical Interview for DSM-IV Axis II Personality Disorders (SCID II). New York: Beiometric Research Department (1994).

[23] First M Spitzer R Gibbon M Williams J Structured Clinical Interview for DSM-IV Axis I disorders (SCID I). New York: Biometric Research Department (1997).

[24] Wittchen HU Zaudig M Frydrich T Strukturiertes Klinisches Interview für DSM-IV. Achse I und II. Göttingen: Hogrefe (1997).

[25] Schneider S Unnewehr S Margraf J Diagnostisches Interview bei psychischen Störungen im Kindes- und Jugendalter (Kinder-DIPS). Berlin: Springer (2009). 10.1024/1422-4917//a000247 23988834

[26] Wittchen HU Pfister H DIA-X-Interviews: Manual für Screeing Verfahren und Interview; Interviewhelft Längsschnittuntersuchung (DIA-X-Lifetime); Ergänzungshelft (DIA-X Lifetime); Interviewhelft Querschnittsuntersuchung (DIA-X-12 Monate); Ergäzungshelft (DIA-X-12 Monate); PC-Programm zur Durchführung des Interviews (Längs- und Querschnittsuntersuchung); Auswertungsprogramm. Frankfurt: Swets & Zeitlinger (1997).

[27] Chisholm D Knapp MR Knudsen HC Amaddeo F Gaite L van Wijngaarden B Client Socio-Demographic and Service Receipt Inventory–European Version: development of an instrument for international research. EPSILON Study 5. European Psychiatric Services: Inputs Linked to Outcome Domains and Needs. Br J Psychiatry Suppl (2000) (39):s28–33. 10.1192/bjp.177.39.s28 10945075

[28] Bock J Brettschneider C Seidl H Bowles D Holle R Greiner W Ermittlung standardisierter Bewertungssätze aus gesellschaftlicher Perspektive für die gesundheitsökonomische Evaluation [Calculation of standardised unit costs form a societal perspective for health economic evaluation]. Gesundheitswesen (Bundesverband Der Ärzte Des Öffentlichen Gesundheitsdienstes (Germany)) (2015) 77:53–61. 10.1055/s-0034-1374621 25025287

[29] Rote Liste Service GmbH Rote Liste 2014. Arzneimittelverzeichnis für Deutschland. Frankfurt am Main: Rote Liste Service GmbH (2014).

[30] Group EuroQoL EuroQol–a new facility for the measurement of health-related quality of life. Health Policy (Amsterdam Netherlands) (1990) 16(3):199–208. 10.1016/0168-8510(90)90421-9 10109801

[31] von der Schulenburg J Claes C Greiner W Uber A Die deutsche Version des EuroQol-Fragebogens. Z Gesundh Wiss (1998) 6:3–20. 10.1007/BF02956350

[32] Ludwig K Graf von der Schulenburg JM Greiner W German Value Set for the EQ-5D-5L. PharmacoEconomics (2018) 36(6):663–74. 10.1007/s40273-018-0615-8 PMC5954069 29460066

[33] Dong Y Peng CY Principled missing data methods for researchers. SpringerPlus (2013) 76(5):484–91. 10.1186/2193-1801-2-222 PMC3701793 23853744

[34] van Buuren S Groothuis-Oudshoorn K mice: Multivariate Imputation by Chained Equations in R. J Stat Softw (2011) 45(3):1–67. 10.18637/jss.v045.i03

[35] Wille N Badia X Bonsel G Burstrom K Cavrini G Devlin N Development of the EQ-5D-Y: a child-friendly version of the EQ-5D. Qual Life Res Int J Qual Life Aspects Treatment Care Rehabil (2010) 19(6):875–86. 10.1007/s11136-010-9648-y PMC2892611 20405245

[36] Perfect MM Turley MR Carlson JS Yohanna J Saint Gilles MP School-Related Outcomes of Traumatic Event Exposure and Traumatic Stress Symptoms in Students: A Systematic Review of Research from 1990 to 2015. School Ment Health (2016) 8(1):7–43. 10.1007/s12310-016-9175-2

[37] Daignault IV Hebert M Profiles of school adaptation: social, behavioral and academic functioning in sexually abused girls. Child Abuse Negl (2009) 33(2):102–15. 10.1016/j.chiabu.2008.06.001 19303636

[38] Nooner KB Linares LO Batinjane J Kramer RA Silva R Cloitre M Factors related to posttraumatic stress disorder in adolescence. Trauma Violence Abuse (2012) 13(3):153–66. 10.1177/1524838012447698 22665437

[39] Shonk SM Cicchetti D Maltreatment, competency deficits, and risk for academic and behavioral maladjustment. Dev Psychol (2001) 37(1):3–17. 10.1037/0012-1649.37.1.3 11206431

[40] Lipschitz DS Rasmusson AM Anyan W Cromwell P Southwick SM Clinical and functional correlates of posttraumatic stress disorder in urban adolescent girls at a primary care clinic. J Am Acad Child Adolesc Psychiatry (2000) 39(9):1104–11. 10.1097/00004583-200009000-00009 10986806

[41] Statistisches Bundesamt [Federal Statistical Office] Bildungsstand [Educational background 2013] Wiesbaden (Germany): Statistisches Bundesamt [Federal Statistical Office] (2017) [cited 2017 8th, march]. Available from: https://www.destatis.de/DE/Themen/Gesellschaft-Umwelt/Bildung-Forschung-Kultur/Bildungsstand/_inhalt.html.

