# Supplementary material for: Health-Related Quality of Life and Costs of Posttraumatic Stress Disorder in Adolescents and Young Adults in Germany
Source: Front Psychiatry. 2020 Jul 15;11:697. doi: 10.3389/fpsyt.2020.00697 (PMC7373788; doi:10.3389/fpsyt.2020.00697)
Supplement: Supplementary file 1 [file DataSheet_1.pdf]

Supplementary material:

Table S1: Unit costs for different resource uses in 2014 Euros

Category	Unit	Price per unit (€)
**Direct resource use**		
**Outpatient sector**		
General practitioner	visits	20.94
Pediatrician	visits	35.85
Dentist	visits	58.33
Orthopaedist	visits	26.54
Gynaecologist	visits	32.46
Oculist	visits	36.31
Dermatologist	visits	19.72
Otorhinolaryngologist	visits	27.56
Psychologist/psychotherapist	visits	81.52
Other medical specialists	visits	40.33
Counselling	visits	103.07
Child welfare institutions	days	81.90
**Inpatient sector**		
General hospital	days	601.28
Psychiatric hospital	days	354.68
Rehabilitation hospital	days	127.22

Table S1 Six-month costs in 2014 Euros by treatment centre (n=87)

	Ingolstadt (n=28)			Berlin (n=27)			Frankfurt (n=32)		
	mean	SE	% of the sample with utilization	mean	SE	% of the sample with utilization	mean	SE	% of the sample with utilization
general hospital	960	399	21	1343	465	22	573	262	13
psychiatric hospital	421	313	7	2444	1298	15	827	775	3
rehabilitation	158	156	4	1026	593	11	221	169	3
outpatient physician treatment	881	145	96	701	139	96	529	128	97
psychotherapy/psychiatric/neurological	555	123	64	326	95	48	330	119	47
outpatient non-physician treatment	7	7	4	50	33	15	5	5	3
Medication	35	15	36	75	22	59	63	25	44
child welfare institutions	2356	969	21	1422	752	19	1192	777	9
Counselling	410	193	25	136	49	44	199	77	44
**Total costs**	**5228**	**1132**	**100**	**7197**	**1953**	**100**	**3609**	**1328**	**100**

Table S3 Six-month costs in 2014 Euros by treatment option (n=87)

	D-CPT (N=44)			wait-list condition with treatment advice (n=43)		
	mean	SE	% of the sample with utilization	mean	SE	% of the sample with utilization
general hospital	848	301	14	1027	314	23
psychiatric hospital	1309	658	11	1085	783	5
rehabilitation	400	249	7	502	325	5
outpatient physician treatment	711	109	98	680	118	95
psychotherapy/psychiatric/neurological	406	82	52	397	105	53
outpatient non-physician treatment	34	21	9	5	4	5
Medication	74	21	52	41	13	40
child welfare institutions	1897	764	16	1374	592	16
Counselling	339	131	39	153	46	37
**Total costs**	**5612**	**1201**	**100**	**4867**	**1259**	**100**
